# Authoritative Parents and Dominant Children as the Center of Communication for Sustainable Healthy Aging

**DOI:** 10.3390/ijerph18063290

**Published:** 2021-03-22

**Authors:** Elizabeth Wianto, Elty Sarvia, Chien-Hsu Chen

**Affiliations:** 1 Department of Industrial Design, National Cheng Kung University, Tainan City 701, Taiwan; 2 Bachelor Program in Visual Communication Design, Universitas Kristen Maranatha, Bandung 40164, Indonesia; 3 Bachelor Program in Industrial Engineering, Universitas Kristen Maranatha, Bandung 40164, Indonesia; elty.sarvia@eng.maranatha.edu; 4 Hierarchical Green-Energy Materials (Hi-GEM) Research Center, National Cheng Kung University, Tainan City 701, Taiwan

**Keywords:** aging population, filial piety, healthy aging, physical activities

## Abstract

The aging population significantly is shifting the center of gravity of the people toward older ages and median age. Indonesia, as one of the most populous countries, needs to prepare for this situation. This study tries to explain whether the elderly’s sedentary lifestyle is the consequence of intergenerational interaction patterns. Filial piety was arguably implemented, as the interaction baseline within a family member affects how the intergeneration communicates. This study uses thematic analysis based on the opinions from 16 respondents’ experiences and values with respect to behavior toward the older generation with a specific inclusion criterion. Sampling structures represented younger-generation adults who interacted daily with the elderly older generation, divided by their marital status, residencies, and living area in Indonesia. Through emerging themes, was is found out that the dominant figure in the family is the communication center in the family. The dominant figure might be an authoritative parent or dominant child. This targeted approach is useful to enhance connectivity within family members, potentially implementing the Internet of Healthy Things (IoHT) for the younger elderly to reduce undesirable sedentary lifestyles and to deliver sustainable healthy aging in Indonesian society.

## 1. Introduction

An aging population is a major global trend for all countries. The aging population is not only increasing in number; it also has a significant impact, with a gradual shift in the center of gravity of the population toward older ages and increasing median age [1]. Developed countries might face demographic changes earlier, but this issue is also inevitable for developing countries. However, adopting what was already set in developed countries might not be suitable because of social, cultural, and technology-acceptance discrepancies. Therefore, Indonesia, as the fourth most populous country, needs to prepare for what might happen soon, as it has already been experiencing an aging population since 2015, when the proportion of the elderly population was more than 7%, a proportion that continues to rise, [2] and the predicted life expectancy in 2019 was 69.44 and 73.33 for men and women, respectively [3].

The UNDP’s vision of sustainable development is to leave no one behind, in a balanced manner, for all segments of society, particularly focusing on the most vulnerable group, including older persons. Together with the World Health Organization (WHO)’s concept of enabling people to be and do what they value throughout their lives, this study will fill in part of the research gap in this area with an exploration of Indonesian cultural perspectives. The WHO’s previous concept of active aging, which was replaced with healthy aging, corresponds more realistically with the health status of most elderly people. A condition free of disease for older adults is considered a utopian ideal; thus, such a condition is not required for healthy aging. In contrast, controlling the health condition to maintain the functional ability that enables well-being has become a more realistic purpose. In line with the argument that it is essential to promote health throughout the entire lifespan so that older people can participate actively in society and enjoy independence and good quality of life [4], physical ability shall ideally be retained as long as possible for older people. 

Chronic disease is prevalent in the elderly population. When it occurs, the progressive deterioration of physical and cognitive abilities makes independent living difficult for the elderly [5]. Such a trend eventually will be the cause of drastic social changes, economic challenges, and new demands to families, communities, and government, including demands in healthcare, whether in formal or informal care for the elderly. These demands and challenges are directly linked to all main dimensions of sustainability—social, economic, cultural, and environmental [6]—as well as the opportunities for the design of the community. To the best of our knowledge, there is still a lack of evidence on how the sedentary lifestyle adopted by the elderly might affect how society reacts properly to encourage the elderly. The sedentary lifestyle, which is broadly defined as the type of lifestyle involving little or no physical activities, is associated with healthy aging and the quality of life, regardless of age or gender [7,8,9].

Nevertheless, even though the elderly fall into the vulnerable group, they already have experience, knowledge, skills, and many non-intellectual factors [10] that contribute to their communities and build up society. Thus, as a sign of respect or being filial, the younger generation might not feel at ease to correct their bad habits; moreover, children may even restrict them from doing some physical activities as the manifestation of the elderly’s previous sacrifice. An explanation of filial piety is given in detail in Section 2, Filial Piety in Indonesia.

Therefore, as part of the ongoing research to enhance the physical activities for the elderly in Indonesia, which has already proven lower than the other comparison communities [11], this study would like to dig deeper in the phenomenon of the sedentary lifestyle, since there are inconsistencies in perceived positive behavior towards exercise. Most of the elderly stated that exercise is important for them. However, they are still reluctant to do it and prefer to stay physically inactive by claiming the lack of companionship in doing exercise as the reason [12]. To get the whole picture that was previously taken from the elderly’s point of view, this study will then look closer from the younger generation’s point of view.

We argue that adopting a sedentary lifestyle in the elderly is a consequence of intergenerational interaction patterns between the elderly and the younger generation. Thus, this study’s main goal is to understand better how intergenerational interaction impacts everyday communication within the family. Therefore, we breakdown the research questions into the following: (1) What is the basic value or ethics that underlie the relationship between younger adults and the elderly? (2) How are basic value or role ethics underlying the relationship between younger adults and the elderly implemented or retained in their knowledge? (3) How and why does the relationship between familial intergeneration differ from non-familial intergeneration? (4) Is it possible to change the value/role ethics? and (5) What aspect could have changed the ideal standard of role ethics to the current practice? The answers to those research questions will fill in the gap and bridge the intergeneration supportive communication to promote sustainable healthy aging for the elderly. 

## 2. Filial Piety in Indonesia

The term filial piety is the central concept in this study. It is well known in Chinese culture and has spread to other Asian countries throughout the Chinese diaspora, diffusing, assimilating, and acculturating with the local values. It is said to be the standard practice towards the elderly who act as parents [13] for what they have sacrificed and provided to the younger generation and is accepted as a reciprocal exchange between generations [14,15]. Respect is a central value in behavior to older generations, and, explicitly in the Asian family, it is often articulated as the sense of obligation to obey, to be courteous, and to respect and care for parents as they age [16]. The persistence of filial value implementation was the cause of this concept chosen as the baseline of interaction between parents and children and how far physical activities are supported, restrained, or modified in Indonesian families and societies.

Briefly aforementioned, this virtue is not exclusively implemented in Chinese culture, as it is commonly found that the family serves as the most important source of informal care and support for older adults. Regardless of the Confucian, Chinese, or Asian traditional teachings, other beliefs also underscore the relations between parents and children. For example, Christianity has the command to “Honor thy father and thy mother: that thy days may be long upon the land which the Lord thy God giveth thee” (Exodus 20:12 King James Version), as the first commandment with a promise, which is emphasized in writing by Paul the Apostle to the Ephesians, “That it may be well with thee, and thou mayest live long on the earth” (Ephesians 6:3 King James Version) [17], and to the Colossians, “Children, obey your parents in all things: for this is well-pleasing unto the Lord” (Colossians 3:20 King James Version). According to the Buddhist moral teaching of good karma, filial piety is analogous to honoring Brahma—the supreme god and the creator of human beings—in Hinduism, as parents have done much for their children [18]. Islam as the major religion in Indonesia and Arabic countries, also provides moral stipulations, which state “Be kind, honorable and humble to one’s parents” (Quran 17: 23), as these commandments mention that people should not say to their parents a word of disrespect and should not resist them but speak to them a noble word. Moreover, a prayer should be said that asks God to be merciful to parents, as they brought children up when they (the children) were vulnerable (Quran 17:24).

These solid intergenerational ties, shaped, structured, and suggested for familial intergeneration support more tangible aspects of affection, such as gestures and manners, tokens, customs, and rituals, as well as asking for trusted advice and obedience [16,17]. The decision to take care of one’s own parents also shape the cultural impacts [19] is perceived as a selfless act, most comprehensive and long-term commitment [20]. Thus, in a broader sense, the practice is supposedly not only done for the sake of parents but also to pay forward good actions and good karma as part of the dharma and social order for a better society [18]. 

However, globalization has forced the younger generation to struggle differently in making a living [17], and raising a family [21]; many of these changes are due to migrating from rural to urban areas and decreasing family size due to the declining average number of children born per woman [22]. Consequently, this intergenerational relationship practice will inevitably change [23].

Indonesian culture has always considered the family as the primary support system for their parents; most older people co-reside with at least one child. It is still considered shameful for a family to admit their parents to a residential aged care facility (*Panti Sosial Tresna Werdha*—Ind) [21]. As a consequence, co-residing with at least one child is preferable as old age security for parents [24] in developing countries [25], even though it does not always correlate positively with the parental health or expectation and is less likely to be preferred by socially active elderly parents [24] until they can no longer be so [26]. Older people are willing to be active as before as long as they have the capability. However, a rigid standard occasionally emerges from the children for feeling guilty and depressed because of their service not being good enough for their old parents [27]. Nevertheless, the previous study showed that the filial piety value is changing; it is now implemented to more tolerant older people [17].

## 3. Materials and Methods

### 3.1. Methods

This study uses a qualitative method based on the respondents’ experiences, meanings, and perspectives [28], as in this case, respondents were independent adults with prior or recent experience of interaction with their parents. The thematic analysis approach used in this study was selected, as this method can identify and interpret patterns in a flexible but specific condition related to experience, the process of interactions, meanings, or values from the respondents’ perspective [29,30].

The data collection technique for this study was to use semi-structured interviews, gathered cross-sectionally from the respondents purposively. Prior to the interview sessions, the researcher conducted the verbally informed consent process, divided into three steps. The first step was to ask for the respondents’ willingness to become a respondent for a broad topic of research, filial piety; the second step was to ask their affordability to do the interview and the approximate time available to do the interview sessions—1–2 h; and the third step was to ask if they were comfortable to have voice notes taken during the interview sessions. All of the data collection process and informed consent used an online service was not directly face to face. To hide the respondents’ identity, all of the names were hidden and initially coded. The semi-structured interviews had three guideline variables, as follows:

1. basic value/norms/role ethics between the respondents towards the elderly;
2. the interactivity between the respondents towards the elderly;
3. differences between the value/norm/role ethics and interaction between the respondents towards the elderly.

All three guidelines variables referred to the implementation between the elderly in respondent’s families (in this case, their parents, grandparents, or close family) and the elderly not in their family, but generally consider the older generation of the respondents. In this study, the youngest age of the elderly is 60 years old, as stated in Indonesian law [31].

### 3.2. Sampling Structures

This study’s respondents were Indonesian Adults with prior experience interacting with elderly parents, and they were sampled using purposive sampling or stratified purposeful sampling. The structure of sampling was as follows.

- Living arrangement: Co-residence and not co-residence with the elderly

The co-residence condition with the elderly was divided decided based on previous reports related to a significant impact on parental health [24,32]. For our study, in order to get firsthand living arrangement condition of the intergenerational co-residencies, we conducted a preliminary survey using a snowball sample technique for Indonesian adults, between 12 November and 10 December 2019. The result showed that adults in Indonesia majority have experience living with an elderly family, or at least have experience in taking care of one. This insight was gathered from 292 adult respondents who stated that 43 people (14.72%) never live with or take care of the elderly; 116 people (39.73%) have the experience of taking care of the elderly but are not living with the elderly anymore; the rest of them (133 people, 45.39%) are still living with the elderly. From the group with the experience of taking care of the elderly (249 people or 85.12% of the respondents), 31 respondents (12.45%) take care of the frail elderly. Thus, by this result, we decided that it would be valuable to get the views from living arrangement co-residencies.

- Area of living: Urban and rural area.

Through its statistical division, the United Nations has stated that the character distinguishing urban from rural areas is not amendable to a single definition for all countries. However, the traditional distinction shows that urban areas usually have a higher standard of living than rural areas. In Indonesia, urban and rural areas are classified based on their administrative jurisdiction. An area is classified urban when its administrative jurisdiction lies in the city of the capital of a regency, while an area is classified rural if it consists of villages (*desa—*Ind) that are located in regencies. The definition of villages in Indonesia is differentiated from the city in terms of being regions with cultivation as a major activity, the way they are managed, where people live, the administration of the governmental service, social and economic activities, and a limitation of minimum 6000 people or 1200 heads of families [33]. In a rural area, agriculture is the type of activity commonly found as an income-generating activity. In contrast with rural areas, urban areas provide a pension after reaching a certain age under the company or governmental regulation. With this arrangement, rural and urban communities have a different mindset of how they view the working activities.

- Major ethnicities in Indonesia: such as Javanese, Sundanese, *Tionghoa* (Chinese Indonesian), Batak, or Balinese people.

In addition to sampling population, this study also implements the following inclusion and exclusion criteria:

- Inclusion criterion: the respondent should be an Independent Indonesian adult, as this stage of age arguably already implements filial piety as part of their role ethics, so the gap in the intergenerational elderly will be more likely able to reach the age of 60. People in this group already have firsthand experience of intensely interacting with the elderly, which act as the older generation of the respondents.
- Exclusion criterion: (1) if the elderly in the family are younger than 60 years old; (2) if the experience was more than ten years ago (the elderly in the family already passed away more than ten years ago), as this would create deviation between experience and memory recollection; (3) if the respondents paid for taking care of the elderly.

### 3.3. Data Analysis

Interviews were done in Bahasa Indonesia (Indonesian Language) and managed with NVivo 12 after transcribed the audio recordings into text, with the following coding process:

1. Transcription in *Bahasa Indonesia.* All of the interviews were done in Bahasa Indonesia and local dialect, and the coding was transcribed verbatim in Bahasa Indonesia to avoid decontextualization.
2. Familiarization of the data and identifying items of potential interest. After transcription, the data were familiarized by the researcher, and if several ambiguities were found, the researcher asked for confirmation on the true meaning of the respondent’s statement and rechecked the transcription with other co-researchers.
3. Classification of the respondent’s demographic information. Demographic classification was predetermined prior to the interviews. Besides the sampling structures differentiating living arrangement (co-residence/not co-residence) and area of living (urban–rural), there was also other demographic classification based on the elderly (relationship, independency, age, financial status, living children, residencies ownership, mobility, supported, and living arrangement of the elderly) and demographic classification based on respondents (age, child status in the family, marital status, socio-economic status, highest education, and sex). A detailed description of demographic data collection is presented in detail in Table 1.
4. Generating initial coding without considering the classification made. Initial coding was generated based on the respondent’s overall expression to represent a broader filial piety context.
5. Categorizing the themes into the 10-item Contemporary Filial Piety Scale (CFPS). The 10-item CFPS is a modified instrument to measure experience and practice of filial piety down from three to two factors: Pragmatic Obligation and Compassionate Reverence. These are proven to be data-driven, efficient, and straightforward, with strong psychometric properties to assess contemporary filial piety with an internal consistency of 0.88 and 0.95 goodness of fit [34]. The constructs were divided into 10 items as follows. There were six items to construct pragmatic obligations: (1) arranging care for parents when they can no longer care for themselves; (2) providing financial subsistence to parents when they can no longer financially support themselves; (3) arranging an appropriate treatment for parents when they fall ill; (4) attending parents’ funerals no matter where one is; (5) visit parents regularly if one is not living with them; (6) being thankful for parents’ nurturing. The four items of the compassionate reverence construct consist of (1) trying one’s best to achieve parents’ expectations; (2) always being polite when talking to parents; (3) trying one’s best to complete parents’ unachieved goals; and (4) always caring about parents’ well-being. By coding participants’ expressions, we would like to make sure that Indonesian society adopts these values. The filled construct gathered by initial coding reveals whether the concept of filial piety is implemented wholly or partially in the Indonesian family and society. Thus, this coding became the first validation of this study; however, it was used by construct to measure the scales. Therefore, we did not ask the participants to fill the instrument.
6. Cross-tabulating categorized answers to demographic information. Cross-tabulating participant’s answers was done based on demographic information to contrast specific expressions or representations generally.
7. Clustering the categorized answers into themes. Themes were generated after clustering the participants’ categorization, so we were able to articulate the construction between the categories into a sound report.
8. Mapping the relations between themes.
9. Drawing conclusions based on unique answers to the phenomenon of the sedentary lifestyle of the elderly and whether or not it is related to how the intergenerational interaction is implemented in everyday life.
10. Translating the related transcription into English for making the report.

This qualitative study interprets the phenomena related to the value or ethical norms that transfer into a practical decision and the underlying reason. Therefore, the process of validating the findings was done based on initial coding into triangulation [35] of the 10-Item CFPS and rechecking with the co-researchers and relevant previous research studies mentioned in the discussion.

## 4. Results

Between April 29th and June 29th, 2020, 14 people (four men, ten women) participated in this study. The interview sessions took 45 to 71 min for each respondent. All of the interviews were conducted one time only with the same respondents. Nonetheless, to ensure data saturation, interviews were done with two more participants (two women) on November 26th, 2020. After coding the findings from 16 participants, the data were saturated [36]. Therefore, the saturation of the data showed sufficient sampling in this study. There is no new information, richness, depth, or diversity of the answers that were expressed by the participants, which were already purposely selected into several demographic representations. Redundant information was received while doing the interview; however, the participants’ expression was careful not to become the basis of the judgment. 

The respondents’ age range was 27 to 52 years old, and they gave responses about a total of 29 familial elderly within the age range of 61 to 90 years old. Among the older people mentioned, six elderly had passed away, and the other 23 were still alive. Based on the intergenerational co-residencies, six respondents shared their residencies with the elderly, while the other ten did not share their co-residencies. However, despite the non-co-residency with the participants, eight elderly shared their residencies with their other children other than the participants, so there were only nine elderly not living in co-residency with their children (four couples and one widow accompanied by a nurse).

Participants represented the younger generation with the residencies of the elderly in a rural area (6 participants to 12 elderly) and an urban area (10 participants to 17 elderly). Five out of the sixteen respondents did not have a spouse (one man, four women), while the other 11 did. All of the respondents are living in the urban or municipal area and represent several major ethnicities in Indonesia (Javanese, Chinese Indonesian *(Tionghoa),* Balinese, Sundanese, Javanese, and Batak). The participants represented the eldest (six participants), middle (five participants), youngest (three participants), and only child (two participants). In this paper, there was no clear distinction of whether the elderly have tendencies to reside with the eldest, youngest, or middle children. Female and single participants in the sampling distribution tend to co-reside with their parents (four participants), excluding one participant for whom the elderly resides without any of their children (one participant). This condition also appears in the overall sampling distribution, with 12 elderly (clustered into seven families) co-residing with their daughter, and eight elderly (clustered into four families) with their son. Thus, this phenomenon shares similarities with the Asian cultural model rooted in Confucian ideology, requiring daughters to provide ongoing daily assistance to their elderly [19]. 

In terms of the elderly, there were six elderly who were financially dependent, and the other six still had income, while the rest were financially independent. However, the financially independent status did not mean that the participants did not give financial support. The majority of the elderly still lived in their own house (13 families), while the other five elderly people (three families) lived in their children’s house. Only one elderly did not have a child and was living in her nephew’s residence, while the other has at least one child (in one family) and the rest of the elderly had two to seven children. However, not all of the children supported the elderly, and five respondents stated that their siblings did not support their parents because of their incapability (i.e., time, living distance, finance, etc.), or because their parents were still independent and wished to be treated the same as when they were younger. From this condition, there were only four elderly who were entirely dependent (mostly because of their health and financial status) and six elderly who were independent, and the majority (19 elderly) were partly dependent on their children or close family. Based on the ability to do Basic Activities of Daily Living (BADL), there were three elderly who could do BADL, while the other 26 others were still able to do BADL independently without any help. A detailed description of the participants’ demographic classification is presented in Table 1.

### 4.1. Ten-Item Contemporary Filial Piety Scale Result 

The aim of using the implementation of filial piety as the primary term in this study was to consider its validity when the transcript corresponds with the constructs to support filial piety as the central concept. The 10-Item Contemporary Filial Piety Scale factors are divided into the following factors structure: pragmatic obligation and compassionate reverence. The first component, pragmatic obligation, contains six-items, while the second component, compassionate reverence, contains four items, as stated in the literature review section.

From the transcript, with the help of NVivo 12, there were 274 references generated or expressed for both of the components, with 134 references generated for pragmatic obligation and 140 references generated for compassionate reverence. The number of references generated from NVivo was not used for quantifying, but for mapping clustered opinions to fit the 10-item CFPS. Each item contains at least seven participants’ expressions, except for “attending parents funeral no matter where one is”, which did not correspond with any of the respondents’ statements. The most expressed opinion for pragmatic obligation was “being thankful to parents for nurturing.” The strong expression shows that they feel grateful for what parents have already sacrificed for participants. Thus, this kindness cannot merely pay for anything. This opinion then corresponds with compassionate reverence for “always caring for parents’ well-being” as the participants’ robust expression. In this item, participants broadly expressed their thought and hopes for their parents, while other items could be translated explicitly into action.

Pragmatic obligation constructs consist of various actions that can willingly be done by the participants; for example, the item “arranging care for parents when they can no longer do so for themselves” was coded in the details of a specific action such as “lift something heavy for her”, to general services, such as “fulfilling their everyday needs”, also including passive activities such as “ready to accompany them, including Sunday” and daily chores.

In contrast with the expression for pragmatic obligation, compassionate reverence represents the values underlying action by the participant, such as “always caring about parents’ well-being”, “giving a good ending (in life) for my father”, “seeing my mom feel happy”, or obeying parents to prevent them from becoming angry, sad, and/or uncomfortable.

The absence of “attending parents’ funeral no matter one is” possibly was missing in this context since the interviews focused on their interactivity value underlying their action or relation towards their parents and the practice of implementing those values. Since almost every construct appears in the respondent’s answers, this shows that Indonesian society still implements and presumes filial piety as the main value to interact with parents.

### 4.2. Categorization and Themes

The transcript was separated into seven categorical answers, described in Table 2. Categories were clustered based on the initial coding, which segmented into more related answers based on participants’ expressions. However, since the coding might be suitable for more than one category because of intercontextual answers, coding can fall into more than one category and subcategory. This study did not count on how many respondents referred to a similar opinion; every opinion is considered unique and thematically representative. 

The first three categories are arranged from a broader to the specific context of the relationship between older and younger generations. In contrast, the other four (fourth–seventh) categories consist of stand-alone opinions that might comprise the ideal relation stated in the first and second categories.

The first category covers the broad context of the interaction between the younger and older generation. These categories express that, in general, the younger generation needs to act less superior to the older generation because of the previous merit of the elderly, as the older generation have already laid a firm foundation for the younger generation as they are now able to become independent. Thus, the younger generation should support the elderly with a declining physical condition. This physical assistance should be implemented for all members of the older generation, not only for the familial one, even though the intensity will be different between these two groups. As the non-familial elderly might have different needs, benchmarks, or habits, helping or interacting with the older generation should consider the overall situation and condition. Equivalents of providing physical assistance to the older generation are not merely because of their declining physical capacities, but more as a symbol of gratitude and that “now is the time” for the younger generation to do the service because they have now become capable and independent. There is no minimum age stated for referring to the older generation as elderly according to the participants. However, the intergeneration always becomes the benchmark for how the younger generation should show a less superior position as a gesture of respect, provide physical assistance and support, and never show disobedience. This finding is arguably based on the stereotyping of the Asian parenting style, with the emphasis on avoiding conflict by the children when disagreement emerges [15]. 

The second category considers the family in a specific context. This category shows how respondents relate their interaction with their parents were based on affordability, decency, emotion, reciprocity, and respect. Affordability, emotion, reciprocity, and respect might be grouped as internal aspects, while decency is more suitably described as an external aspect. However, many participants do not dare say that reciprocity is how they perceived why they would like to treat their parents due to their previous merit. Besides reciprocity, some participants instead actions are a token for respect and emotional bond. Affordability also appears in several manifestations. Finance, time, and knowledge are the most mentioned factors they mentioned during the interviews. The only external aspect shows that there is decency toward their action, which is able to judge externally. This aspect is arguably related to Asian collectivist culture, as they may reckon that society is judging their behavior. 

The third category is familial scope. This category expresses more concrete action than the second category and explains why, how, and when respondents interact. In this category, the point of view of how children support their parents can be divided into two types of benefit. The first benefit is for parents, and the other is for children. It was found that not every action done by the children is for the sake of parents. They also act to fulfill the standard as an excellent child (as they were already trained for it), to prolong the elderly’s good condition so that they still have time to get the elderly’s advice and moral support. It is hard for children to have sick parents because healthy parents are easier to support. Limited time to give service to the elderly was also a popular opinion from the participants. This expression might correlate with “attending parents’ funeral no matter where one is” from the 10-Item CFPS scale, which was never expressed by any of the participants. The younger generation in Indonesia arguably focuses more on living parents. The expression, as mentioned earlier, for the younger generation might relate to how the ethical value firmly holds.

The fourth category was to explain the baseline of the ethical value previously being taught. Surprisingly, multiethnic Indonesian, which is separated by ethnicities, customs, religious beliefs, and rites, although bound by Pancasila as this country’s foundational philosophy, refers to supporting parents as mandatory. However, the deeper reason children would support their parents may vary according to their internal or external motivation. 

Some respondents are driven by external motivation: “Asian family will prioritize family”, “it is just common sense in communal society to do so”, or “showing well manner to keep my family dignity.” The internal motivation, which is driven by personal value, varies more, according to factors such as Christian beliefs (“reap what you sow,” “eldest right,” “honor you parents”), Islamic beliefs (“paradise is beneath mother”s feet,” “doing good to others”) or others expressions, e.g., “belief that our parents already give their best”, “pride for my parents”, and “this is how I trained.” Exclusively doing the service toward parents also appeared for some answers, as they believe that supporting their parents is private, familial things; if an “outsider” interrupts, then it might be a presumptuous action and may jeopardize the family harmony.

The fifth category expressed rationalizations or reasons why the action in the third category was consistently performed by the respondents. Aside from general reasons such as affordability and moral reasons, as stated in the second and fourth categories, these categories explain why children might appear ignorant to their parents for some reason. Strong expressions fell into some judgments related to children’s life partners, who might uphold different values, or the tolerance between married siblings to support parents. The previous expression’s complexity also increases if respondents’ siblings were the ones with whom parents co-resides with a state of chronic disease. Simultaneous treatment for an extended period is why non-co-resident children are reluctant to interfere with the decision made by co-resident children. 

The sixth category responds to how the respondents, as the younger generation, feel guilty to their parents. These feelings might appear if they failed their relationship base, as stated in the second category. It is expressed that even if the request was fulfilled, the attitude and attribute for doing it should also be right. There would be no meaning to doing the right thing if the children delivered it wrongly. This expression shows that Indonesian people are already used to bowing to their parents to keep balance and familial harmony. In order to maintain harmony, some answers revealed that obeying parents is the easiest way to do it. There are many factors that might differentiate one family from others, but the more authoritative and independent the parents, the more that children become subordinate, and vice versa. 

The seventh and last category reveals the opinion related to physical activities for the older generation. The majority of participants agreed about not allowing their parents to do labor or chores. The younger generation strongly expressed that the advanced stage of life is the stage to “take a good rest.” However, according to the Physical Activities Score for the Elderly, the classification of older people’s activities is divided into activities correlated with recreation, sport, housework, and working activities. Nevertheless, restriction to one type of activity might reduce the parents’ overall physical activities, as they are forbidden from doing chores, outdoor gardening, or farming (especially for the ones who lived in the rural area) but were allowed to nurture grandchildren.

An effort to contrast answers by demographic categorization was made using a cross-tabulating technique. Demographic categorization mentions were above 17 (seventeen) points. Nevertheless, the respondent’s answers showed high variation, and most answers could not be contrasted. However, the following two findings were extracted. 

First, the elderly who lived by themselves (did not co-reside) with any of their children seemed to decrease the children’s expression of feeling guilty for not providing better conditions for the elderly. This “better” condition can be interpreted as independence in financial status or the elderly’s ability to do the basic activity of daily living (BADL), including personal hygiene, dressing, toileting, transferring, and eating. In this context, the respondents exclude the expression of “supporting” the elderly with day-by-day tasks. Extending this point to some degree, a separate place of living will arguably reduce the level of direct observation of the elderly, but in contrast with other research, this condition had significant adverse effects on the elderly’s physical health, cognitive ability, and psychological health [37].

Second, all of the respondents argued that as filial children, they supported their parents despite any condition the elderly had. This contrast is related to all of the expressions they provided for their parents as they got older. Nevertheless, they also mentioned that not every sibling had done so. This situation shows that filial piety practice is mandatory for the communal Indonesians among the younger generation and children. However, this practice comprises many aspects, including the subjects’ personality, living environment, collectivistic culture, and intergeneration co-residence. In addition, the subjects involved here included siblings’ personalities as the other younger generation on the family. Unquestionably, filial piety was expressed in general answers for the respondents to be filial. Thus, seniority issues in a broader context (e.g., organizational culture) continuously occur. 

In some cases, the older generation still has its full independence and usually tries to retain this condition as long as possible. In most cases, the ability to be independent is accompanied by their good health condition, financial freedom, ability to do their everyday tasks independently, and refusal to be pitied by their children because of the declining physical state. The power of authoritarians also affected unmarried children more, whether they shared their residencies or not. This is arguably because their parents are still their only nuclear family. The descriptive findings are similar to other related contexts, known in parenting style as authoritative parenting [38]. 

On the contrary to the authoritarian parents, there is an emerging younger generation who stands out from their siblings, outpower their parents, claim their independence as an adult, identify with the role of the advisor in the family, most of the time hold important social roles in the community, and to some degree tend to show support in terms of financial benefactor for both their siblings and parents. This typical personality of adult children in this study is defined as dominant children. On more than one occasion, this type of personality claimed the authority to act on behalf of the parents and identified, by official or unofficial status in the family, as the oldest or most successful, or in co-residence with the parents, hence regularly taking control of vital decisions, including the decisions made for their parents with less power. Therefore, exclusively for the familial intergeneration relation, it is found that there were tendencies to have a more substantial influence on the family based on their expression and contrast. In this study, these characters or roles are defined as the “family leader.” 

From this point onwards, interactional patterns between younger and older generations for discussion in this study focus on the role mentioned earlier. As the older generation, authoritative parents still play a role as the leader in the family, even though children become adults and independent. This personality of authoritative parents can repress children’s freedom to speak up. Obedient children will mostly stand by and see from afar, since the chance to interfere with parents’ decisions is low. Expression from the children of authoritative parents sounds passive: “I am doing what is regular because they don’t want to be specially treated and they don’t want to be pitied, so we act as usual; nevertheless I always stand by. It should be that they asked first, then I offer my help; otherwise my father won’t like it.” Fortunately, in this family, both of the parents are still alive, and although the elderly have a comorbid disease, they were able to manage and overcome the disease through the best medication and physical treatment. However, not every family has this kind of mindset.

As the second type of leader, dominant children frequently come out with their position as the family’s decision-maker, straight to what they think is the best way out, even if other family members do not feel at ease with it. For example, here is a typical expression: “honestly, our communication (with my father) is not well, it is getting better since he got sick… as the matter of his decision, I gave him some experience for he knows the risk”; another participant stated: “In several of times, my mother cannot be accompanied, I am not feeling guilty for this, because I have a more important thing to do.” This expression does not appear if the parents hold the leader’s supremacy. Another expression related to the authority to support parents was as follows: “For me, the family is built to support each other, it is exclusively for family, not for an outsider, the filial concern might easily disappear if the spouse is not right.” All of the expressions, as mentioned earlier, sound bold and decisive from the dominant children. 

In contrast with the dominant children, the respondents’ expression as the dominant children’s sibling might sound more passive: “I have several adult siblings, everyone has their own opinion, and since I am not the dominant voice, I follow the others. Also, my father lives in co-residence with my sister. If I impose my will, then maybe my sister’s family will be troubled.” No doubt, boldly placing their opinion and financially supporting their less dominant parents and siblings are the way the dominant children show that they care.

Unfortunately, this condition might lead to a wrong turn if the dominant voice is from someone who lacks knowledge or attention, as they might direct the family in the wrong direction. For example, one participant expressed, “My feelings toward my parents are hard to describe, as we are not from wealthy family, to support me and my siblings’ education is not an easy job. Therefore, I like them to stop working (at that time), but I made mistakes by my lack of knowledge because I only notice after they quit their job, my mother’s health condition is declining. I regret my previous decision and would like to reverse that action by telling her to walk every day.” Unfortunately, dominant children might not be aware that the family’s dialog is leaning towards their dominancy or thinking it is right.

Henceforth, triggering the leader’s attention towards physical activities might increase the sustainability of healthy aging for the elderly. The two leaders’ details clustered into strengths, weaknesses, opportunities, and threats based on the extracted coding illustrated in Table 3 and Table 4.

## 5. Discussion

The acknowledgment that the “family leader” acts as the agent of change to drive the nuclear and extended family may become an opportunity to achieve sustainable healthy aging. The fact that authoritative parents are still able to do routine physical tasks and determine their life indicates they are using their crystallized intelligence, the type of intelligence for maintaining habits; retain their intrinsic motivation and self-determination as they demand equal treatment from the environment; and still have a positive indicator of well-being, namely meaning in life, life satisfaction, positive affect, and self-esteem, which are negatively associated with depression and apathy. Less life satisfaction also occurs when the elderly—especially women—live with their spouse instead of their adult children [39] or live alone [40]. Fortunately, all aforementioned factors correlate with no sign of cognitive declining or cognitive impairment as decline in cognitive performance is arguably associated with declines in attentional, routine physical tasks, as well as a state of frailty [41]. Financial freedom for the authoritative elderly does not mean that they are not categorized as entirely independent from their children, as they still need some technology-related advice. Nevertheless, in this case, the authoritative elderly are perceived by their children as not hoping to receive the financial transfer from their children as is usually arranged in Asian culture [42], which is more strongly related to the non-coresident children’s characteristics [43]. Despite the parents’ desire to get a financial transfer or not, in our case, the majority of the parents wish to live in their home. Thus, the migrated children would not co-reside with their parents. 

In terms of intergenerational co-residence in developing countries and the characteristics of the first family leader, there are tendencies to have the older generation as the head of the families; therefore, the condition is consistent with the findings that most parents were living in their own house. This type of family is more traditional. The elderly–—mostly men—typically retain power and authority until advanced age through control over the property. This condition is persistent and does not decline in developing countries, as the younger generation relies more on the older generation for housing and the prospect of eventual inheritance [25]. 

We argue that the best option for this type of leader is to trigger them to understand the specific positive indicators of well-being and avoid the negative well-being indicator for the elderly [41]. Opening up the idea of exploring their self-discovery of what they have not done during one’s youth could also be a feasible option [44]. In accordance with the suggestion above, the children should be able to have some checklist to double-check their parent’s latent health condition, because age is still the strongest known risk factor for some diseases [45].

Dominant children, as the second type of family leader, mostly appear in the form of people who migrate from rural to urban areas. Migration is a typical demographic behavior in developing countries, including Indonesia, which has a significant impact on migrant household welfare. Migration decisions were based on the concrete prospect for increasing income, better education, living conditions, and employment opportunities [46]. Initially, participants who migrated from rural areas have the characteristics of the ones who pursue education and likely remain in the cities and seek work after completing their education. This strategy has proven useful for getting higher income afterward [47]. Accordingly, the affordability of financial support is combined with their boldness to put forward their opinion in the family, which is then more likely approved by others in the extended family. Hence, the decision to become a benefactor for their extended family is needed because, as a developing and large populous country, Indonesia does not yet have steady health care facilities [48] nor social care service to support long-term care of the older people. Therefore, the feasible practice of not abandoning parents as a form of intergenerational gratitude [49], or even extending this to non-familial elderly, is a developing moral stance. Nevertheless, discussing elderly caregiving means involving their adult children’s spouse. With new roles as husband or wife, in-laws might have different priorities, resources, taboos related to elderly gender differences (e.g., wife to father-in-law and vice versa), poor health condition, and another cultural context, which might increase the complexity [50]. Our data show that all married participants who migrate from rural to urban areas still live in urban areas and leave their elderly behind. The decision to migrate was possibly made when the elderly’s health condition was better, or when the elderly had not reached the age of 60, as the decision to migrate was affected by parent’s health [51]. By the time the older generation become aged, the children already set up a family in the urban area and have different circumstances to manage their living arrangement. Economic benefits of living in the urban area also enable them to give financial transfer, which becomes the reason they are willing to respond.

Having the advantages of being obeyed by the extended family, then, the two dominant figures aforementioned will be beneficial as the center of the communication. Triggering the dominant figures to initiate familial communication will arguably be effective as a way to activate other members. However, the source, channel, and medium might be based on their literacy, trust [52], and affordances [53]. Nevertheless, the simultaneous interplay between the medium and the receiver creates a different way to communicate. Fortunately, computer-mediated communication in the form of smartphones with an internet connection is arguably familiar for baby boomers and younger populations. Based on the polling conducted by Indonesians polling 5900 samples, the percentage of internet usage in Indonesia in 2019 is 64.8% (171.17 million out of 264.17 million residents), with the distribution of users 55–59 years old being approximately 2.5 times higher than that among those 60–64 years old [3]. Therefore, smartphones are considered as a suitable communication channel in bridging the interaction between the younger and older generation in Indonesia. Interaction using the Internet of Healthy Things (IoHT) [54] might be affected by a reluctance to create effective communication, but feasible [55] once the habit of the family leader is recognized. Therefore, considering the family leader’s preferences to start the interaction and the needs for social engagement in their daily lives, brings us to the idea of enhancing the connectivity of each member in the family with the use of the internet usage with the embedded platform to the specific theme related to sustainable healthy aging maintenance.

This paper discusses the perspectives of a limited number of participants residing in Java Island as the selected socio-demographic of independent Indonesian adults representing the younger generation. In this study, the primary cluster was divided into an area of living and co-residencies with the attributes that enrich the opinion of each respondent. These attributes are presented in Table 1. There is also a limitation in the opinion related to the specific nurturing process of sick elderly with gender differences between older and younger generations. Thus, there is a possibility that the exploration might potentially expand as Indonesia consists of many ethnicities and is bound by religion as their way of life. Thus, the study mostly focuses on the participants in the Java area—the most populated, aging population, and Indonesia’s designated migration island. 

## 6. Conclusions

**Figure 1:**
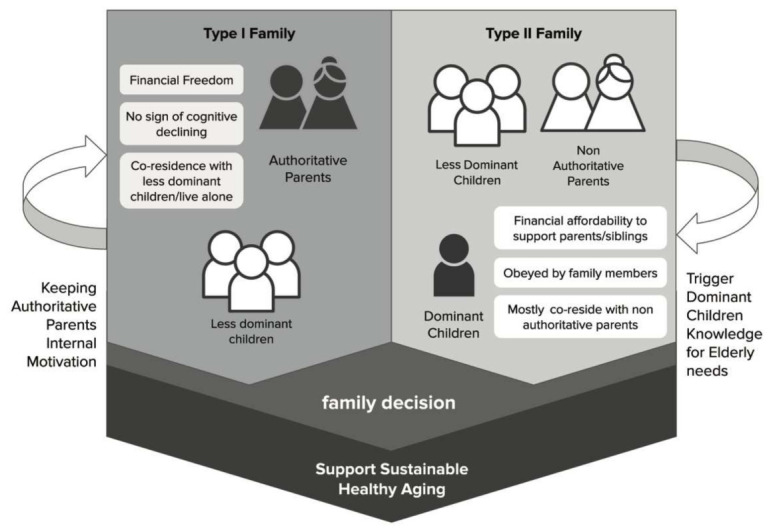
Communication model to support sustainable healthy elderly in intergenerational interaction.

The inevitable decline in physical abilities in older age has a multidimensional point of view. Thus, the adult children’s responsibility to monitor their parents’ condition while performing in their professional field and in their own family should be anticipated. Both authoritative parents and dominant children need some trusted guidance to run within the family’s communication. This creates an opportunity to increase awareness, interest, and reminders for their parent’s well-being. Therefore, exploring the details the following is essential to support the actual implementation of healthy aging to reduce sedentary lifestyle: (1) how the communication medium is used by both parents and children in everyday life, (2) internal motivation of authoritative parents, and (3) triggering the action of the dominant children. The model for supporting this idea is presented in Figure 1.

Considering the details aforementioned and then applying technological-based design (e.g., virtual assistant), or any regulation arrangement to retain the elderly’s physical and cognitive state, is feasible as long as the unique intellectual factors that influence older adults’ learning ability are taken into account [56]. The feasibility of this implication is supported by the elderly’s positive behavioral change or the concept of Cattel’s Crystallized Intelligence (Gc) correlated with wisdom. These concepts also include the ability to view the problem from multiple perspectives, accept compromise, and recognize the limitation of knowledge [57]. As the possibility of enhancing and maintaining this type of intelligence is through education, then further research on how to train, measure, and engage the interaction through a familiar type of IoHT is promising for Indonesian elderly society.

## Figures and Tables

**Table 1:** Classification of participants’ attributes.

No	Classification Attributes	Class	Description
1	Living area	rural; urban	Living area of the elderly.
2	Elderly relationship with the respondents	parents; parents-in-law; unmarried aunt; and grandparents	Elderly relationship with the respondents.
3.	Elderly dependency	independent; partly dependent; and totally dependent	Dependency status of the elderly according to the respondents.^a^
4	Elderly Age	66–65; 66–70; 71–75; 76–89; 81–85; and 86–90	Elderly age range.
5	Elderly financial status	financially independent; still have income, and dependent	Financial ability of the elderly.^b^
6	Children of the elderly (number)	1; 2; 3; 4; 5; 6; and 7	Relates to how many children alive they still have.
7	Elderly co-residency status	with children; without children ^c^	The co-residency status is not always with the respondents.
9	Elderly mobility	able to do independent mobility; not able to do independent mobility	Mobility capacity of the elderly.
10	Elderly supported by	all children; available/willing children; and no support	Related to who supports the elderly.^d^
11	Respondents’ living arrangement with the elderly	respondents living arrangement with the elderly: co-residency; and non-co-residency	It is separated by the number 7 (elderly co-residency status).
12	Respondents’ age range	26–30; 31–35; 36–40; 41–45; 46–50; and 51–55	Age range of the respondents.
13	Respondents’ child status in the family	only child; eldest; middle; and youngest	Respondents’ child status in the family.
14	Respondents’ marital status	married; un-married	Respondents’ marital status.
15	Respondents socio-economic status	1 to 10 (1 for not enough, 10 for more than enough)	Subjective socio-economic of the respondents.
16	Respondents’ highest education	Bachelor’s degree; Master’s degree; doctoral degree.	Highest education achieved by the respondents.
17	Respondents’ sex	female; male	Respondents’ sex.

^a^ This dependency does not always mean that the children do not/are not able/are not willing to support the parents. ^b^ This status does not mean that the children do not support the elderly, but refers to the elderly’s capacity to financially support themselves according to their children. ^c^ This item clearly states whether the elderly live in co-residence with the respondents or not. ^d^ This item refers to whether they are supported by all children, not supported, or supported by the available children. The support is not only related to financial, but also to other aspects.

**Table 2:** Categories clustered based on initial coding.

No	Categories	Description	Subcategories
1	Related to older people (in general)	Opinion in a broader context related to how younger and older generations should act. In these categories, older generation includes non-familial elderly.	Brain activation for the elderly; judgment of elderly’s physical condition; societal power through blood relation; parents cannot compare with anyone; independent and capable younger people; younger people needs to act less superior towards the elderly
2	Relationship between parents—children	Opinion related to how the respondents in their role of children state their relationship with their parents exclusively.	Affordability; decency; emotionally; reciprocity; respect
3	Children’s supported to	Opinion related to how, when, and why the respondents support their parents	How; when; why.
4	Factors influence relationship	All of the factors can comprise the baseline of their thought and act.	Cultural value (including religious value) and personal value
5	Respondents’ point of view	Specific opinion regarding anything explaining their relationship to supporting their parents. Some sort of reason on how they thought or act towards their parents.	Blessing from the elderly; changing priority; I do what I like people do to me; I will be disappointed if I can’t do it; reason to keep the ideal norm; appropriate to pay respect to parents; and tolerance between siblings
6	Wrongdoing to my parents	Specific opinion regarding respondent’s guilt for the action they did to their parents.	I’m not doing what supposed to do; I don’t feel like (to do) it; I don’t have enough knowledge before; I failed the manner; I failed to calm him down; I failed to give what best for him; I lied to them; I think that it is not important; and my parents think differently
7	Related to elderly physical activities	Specific opinions from the respondents related to physical activities of the elderly people.	because they are old; they need to have rest; disease; the elderly are not supposed to/not allowed to do it; full dependency of the elderly; physical assist for those who need it (not only to elderly); physically independent

**Table 3:** Authoritative parents’ characteristics.

No	Item	Description
1	STRENGTH	Financially independent Advantage of being obeyed by a family member Able to motivate themselves
2	WEAKNESS	Children do not dare to argue with parents even though there are other good points of view from their children Gradually cognitively declining
3	OPPORTUNITY	Positive indicator of well-being (like their life to go on as the usual condition), makes it possible to increase self-determination
4	THREAT	The elderly depend on themselves to measure their capacity of doing daily living activities

**Table 4:** Dominant children characteristics.

No	Item	Description
1	STRENGTH	Advantage of being heard by family members. Take the exclusivity of taking care of parents as their privilege. Affordability in terms of financial state to support parents.
2	WEAKNESS	The false assumption that they should make the elderly stop working as a reward for previous merit. Intensity to take care of parents is determined by their priorities. The authority to take care of parents strictly within family members.
3	OPPORTUNITY	Feel sad about the elderly’s declining condition, which makes them take care of parents. Feel obligated to “repay” parent’s merit. Limitation of time to take care of the parents.
4	THREAT	Neglecting parents when they feel it is not important. Sway of priorities if their spouse has different priorities towards taking care of parents. Difference of cultural perspectives between parents’ families with their own family; for example, the spouse’s cultural perspective might not be suitable to approach parents’ well-being.

## Data Availability

The data are not publicly available due to privacy issue.
